# Effects of human papillomavirus (HPV) type 16 oncoproteins on the expression of involucrin in human keratinocytes

**DOI:** 10.1186/1743-422X-9-36

**Published:** 2012-02-14

**Authors:** Eszter Gyöngyösi, Anita Szalmás, Annamária Ferenczi, József Kónya, Lajos Gergely, György Veress

**Affiliations:** 1 Department of Medical Microbiology, Medical and Health Science Centre, University of Debrecen, 4032 Debrecen, Nagyerdei krt. 98, Hungary

**Keywords:** HPV 16, Oncogenes, Keratinocyte differentiation, Involucrin

## Abstract

**Background:**

The human papillomavirus (HPV) life cycle is closely linked to keratinocyte differentiation. Oncogenic HPV infection has been shown to hamper the normal differentiation of keratinocytes; however, the underlying mechanisms responsible for this phenomenon are yet to be clarified. Here, we aimed to study the effects of HPV16 E6 and E7 oncogenes on the expression of involucrin (IVL), an established marker of keratinocyte differentiation, in human foreskin keratinocyte (HFK) cells.

**Results:**

The differentiation of HFK cells by serum and high calcium significantly increased both the mRNA and the protein levels of IVL. The E6 and E7 oncoproteins of HPV16 together caused strong down-regulation of IVL mRNA and protein both in proliferating and in differentiating HFK cells. To study the effects of HPV oncogenes on the *IVL *promoter, we made transient transfection assays and luciferase tests and found that HPV 16 E6 but not E7 repressed *IVL *promoter activity in proliferating HFK cells. The inhibitory effect of HPV 16 E6 on the human *IVL *promoter could be localised to the proximal regulatory region (PRR) of the gene.

**Conclusions:**

These results suggest that the down-regulation of *IVL *promoter activity by HPV 16 E6 significantly contribute to the inhibition of endogenous *IVL *expression by the HPV 16 oncoproteins. In contrast, the down-regulation of endogenous IVL expression by HPV16 E7 is probably not caused by a direct and specific effect of E7 on the *IVL *promoter.

## Background

Papillomaviruses are small DNA viruses, with a circular double-stranded DNA genome of about 8 kbp length [1]. Over 100 human papillomavirus (HPV) types have been identified until now, of which about 40 is able to infect the genital mucosa [2]. Low-risk HPV types (HPV 6, 11, 42) are mainly found in benign genital lesions (condyloma acuminatum) or low grade cervical dysplasias, while high-risk or oncogenic genital types (HPV 16, 18, and others) are causally linked to the development of cervical cancer [3].

The E6 and E7 oncoproteins of high-risk HPVs are responsible for the transforming activity of the virus [4]. High-risk HPV E6 induces the degradation of the p53 tumour suppressor protein through the ubiquitin-proteosome pathway [5]. In addition, HPV E6 is able to bind several other cellular proteins, some of which can mediate transforming activity independently from the p53 pathway [5]. High-risk HPV E7 is able to bind to the pRB (retinoblastoma) tumour suppressor protein, resulting in the functional inactivation and degradation of pRB [6]. By binding to pRB/E2F complex and, by releasing free E2F transcription factors, HPV E7 induces the progression of the cell cycle [6].

The life cycle of human papillomaviruses is closely linked to keratinocyte differentiation. HPVs initially infect proliferating basal cells of the squamous epithelium, while virus production is associated with terminally differentiated layers [7]. The cellular DNA replication machinery is reactivated by the E7 oncogene in differentiating keratinocytes to provide a cellular environment that is permissive for the replication of the viral genome [7,8]. This activity of HPV 16 E7 was shown to delay the induction of the keratinocyte differentiation markers involucrin and keratin 10 [9].

During the multi-step process of keratinocyte differentiation, the expression of genes involved in the process (such as keratins, transglutaminase 1, involucrin, etc.) is tightly regulated. The presence of HPV 16 E6 oncogene was shown to hamper the normal differentiation of keratinocytes induced by serum and calcium or by normal stratification in organotypic cell culture [10-12]. However, the underlying mechanisms responsible for the perturbed differentiation of keratinocytes by the HPV oncogenes are only partially elucidated.

It is reasonable to assume that the E6 and/or E7 oncogenes may have an effect on the transcription of key cellular genes involved in the differentiation of squamous epithelium. Indeed, HPV 16 E6 was shown to modulate the expression of several differentiation-associated genes in human foreskin keratinocytes [13]. Using a xenograft model, Lehr and co-workers showed that infection of human keratinocytes by certain HPVs (type 11 and 59) causes altered expression of certain CCE (cornified cell envelope) proteins, such as loricrin and small proline rich proteins (SPRR), both on the mRNA and on the protein level [14,15]. However, it is not known whether the HPV oncoproteins have effects on the promoters of differentiation-regulated genes or exert their effects post-transcriptionally.

The aim of this study was to investigate the effects of HPV 16 E6 and E7 oncogenes on the expression of the involucrin (*IVL*) and transglutaminase 1 (*TG1*) genes, which are established markers of differentiation of squamous epithelium. IVL protein is a 68 kDa, rod-shaped molecule containing several glutamine residues. It is found in the cytoplasm and cross-linked to membrane proteins by keratinocyte transglutaminases in differentiating keratinocytes [16].

Here, we found that the HPV oncogenes down-regulated both *IVL *mRNA and protein levels in human foreskin keratinocyte (HFK) cells. In order to study the molecular mechanisms that are responsible for the gene expression alterations by the HPV oncoproteins, experiments were performed using HPV 16 E6 and/or E7 expression plasmids along with luciferase reporter constructs containing parts of the regulatory region of the human *IVL *gene.

## Results

### Generation and characterization of human foreskin keratinocyte (HFK) cells expressing HPV 16 oncogenes

**Figure 1:**
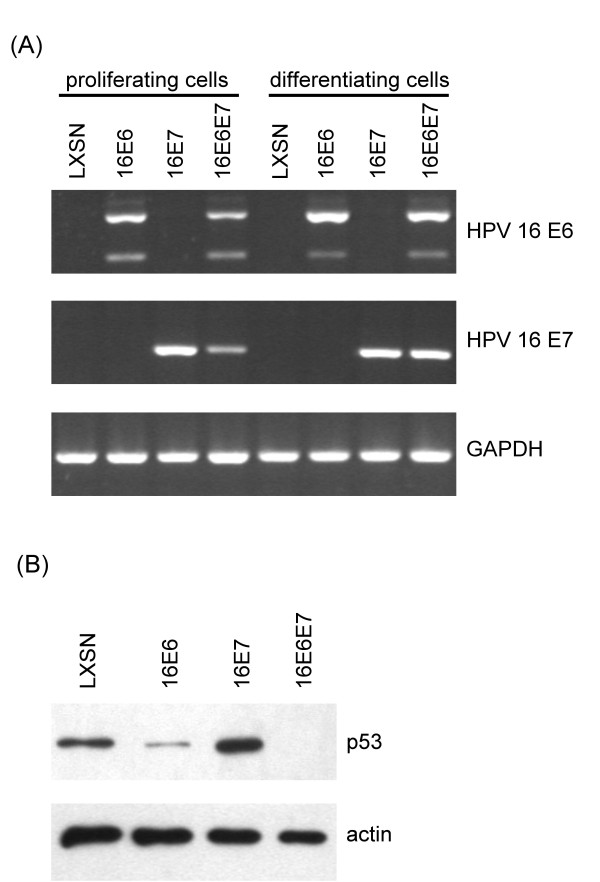
**The expression of HPV oncogenes in transduced human foreskin keratinocyte (HFK) cells**. (**A**) Cells were transduced by recombinant retroviruses carrying either the control vector (LXSN) or vectors encoding HPV16 E6, E7, or E6/E7. The cells were either left in serum-free medium (proliferating cells) or induced to differentiate in DMEM (with serum and high calcium). RT-PCR analysis was used to confirm the expression of the appropriate HPV oncogenes in the different cell lines (GAPDH was used as an endogenous control). (**B**) The effect of HPV16 E6 and E7 on the level of p53 protein. Protein extracts from transduced cells were subjected to Western blot assay for p53 or actin (the latter as loading control). Parts of representative X-ray films are shown.

To study the effects of HPV oncogenes on the expression of cellular genes, human foreskin keratinocyte (HFK) cells were transduced by recombinant retroviruses carrying either the control vector (LXSN) or vectors encoding HPV16 E6 or E7 or both oncogenes. The presence of HPV E6 and/or E7 mRNA in the transduced cells was demonstrated by RT-PCR (Figure 1A). The 2 PCR bands shown on Figure 1A (upper panel) represent the full length E6 mRNA and the E6*I spliced variant, in accordance with previous results [17]. The presence of functionally active HPV oncoproteins was confirmed by demonstrating their effect on the level of the cellular p53 oncoprotein (Figure 1B). As expected, transduction of HPV16 E6 resulted in a decreased level of p53 protein (about 10% of that seen in control transduced cells, as indicated by densitometry), while expression of HPV 16 E7 led to the stabilization and thus higher level (1.6-fold increase) of p53 protein [5,6].

**Figure 2:**
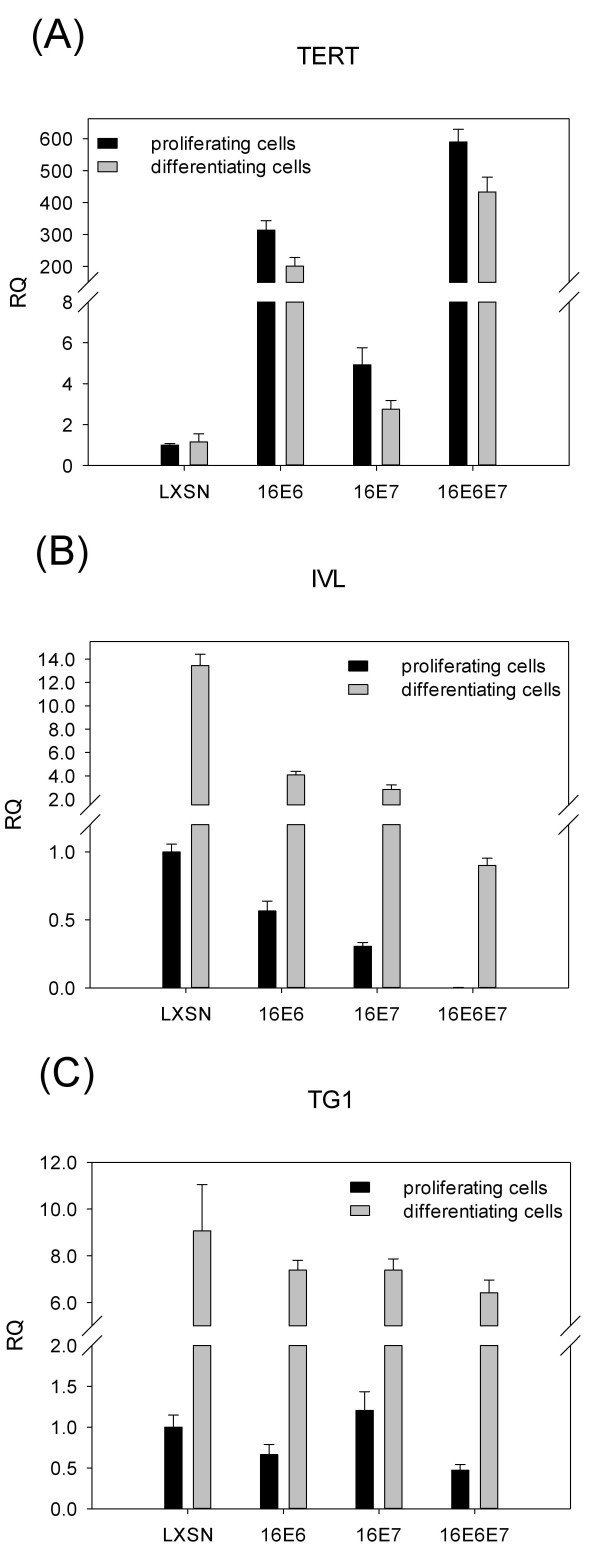
**The effect of HPV oncogenes on the mRNA levels of selected genes in HFK cells**. Generation of cell lines and induction of differentiation was performed as described in the legend to Figure 1. Relative Quantification (RQ) values were obtained by real-time RT-PCR analysis to see the effects of HPV 16 oncogenes on the level of TERT (**A**), IVL (**B**), or TG1 (**C**) mRNA in HFK cells. The RQ value of proliferating cells transduced by LXSN was set to 1, and other values are shown relative to this. Each reaction was performed in triplicate at least three times. Representative graphs are shown, with error bars indicating standard deviation.

The effects of the HPV oncogenes on the endogenous mRNA levels of different cellular genes were studied using real-time RT-PCR assays. In these experiments, cell lines were used within 5 to 6 passages after transduction, as we wanted to see the acute effects of the HPV oncogenes on the expression of the cellular genes. The transduced cells maintained the proliferating, non-differentiating phenotype when left in serum-free medium, while differentiation was induced by changing the culture medium to DMEM (with serum and high calcium) for 24 h, in accordance with the results of previous studies [9,11]. An established function of the HPV 16 E6 oncogene is the transcriptional trans-activation of the human *TERT *(telomerase reverse transcriptase) promoter [18,19]. Accordingly, the presence of E6 led to a considerable (about 300-fold) increase in the level of *TERT *mRNA (*p *< 0.001), while E7 had a weaker but still significant (*p *< 0.05) effect (Figure 2A). As expected, induction of differentiation had no significant effect on the level of *TERT *mRNA in any of the transduced cells.

### Effects of HPV 16 oncogenes on the expression of selected cellular genes involved in keratinocyte differentiation

Real-time RT-PCR assays were used to examine the effects of the HPV oncogenes on the expression of the squamous differentiation marker transglutaminase 1 (*TG1*) and one of its major substrate involucrin (*IVL*) in HPV oncogene transduced cells. As expected, induction of differentiation of HFK cells by serum and increased calcium resulted in highly increased levels of both *IVL *and *TG1 *mRNA (*p *< 0.005) (Figure 2B and 2C). In proliferating cells, both E6 and E7 had significant inhibiting effect (*p *< 0.01) on IVL mRNA levels (Figure 2B). We found a very strong down-regulation of *IVL *mRNA in cells expressing both HPV oncoproteins (HFK-16E6E7) compared to vector transduced (LXSN) cells (*p *< 0.001). Both E6 and E7 caused significant (*p *< 0.001) down-regulation of *IVL *mRNA in differentiating HFK cells. Again, the strongest effect was seen in cells expressing E6 and E7 together (*p *< 0.001). In proliferating cells, HPV 16 E6 and E7 oncogenes together had only moderate inhibiting effect on the endogenous mRNA level of *TG1 *(*p *< 0.05), while in differentiating cells, the HPV oncogenes had no significant effect on *TG1 *mRNA expression (Figure 2C). These results suggest that the effect of the HPV oncogenes on IVL expression is either more direct or more specific than that on TG1 expression.

**Figure 3:**
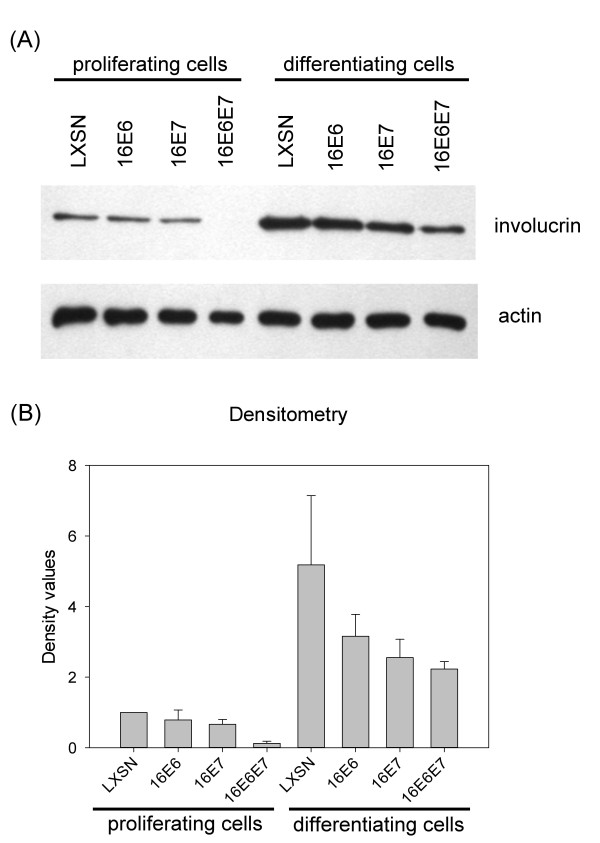
**The effect of HPV oncogenes on the level of IVL protein in HFK cells**. Generation of cell lines and induction of differentiation was performed as described in the legend to Figure 1. Ten μg of protein extracts prepared from the indicated cell lines were subjected to Western blot analysis for IVL or actin protein (the latter as loading control). (**A**) Parts of representative X-ray films are shown. (**B**) The amount of IVL protein was quantitatively analyzed by densitometry and standardized to actin protein level. The standardized density value of cycling cells transduced by LXSN was set to 1, and other values are shown relative to this. Values show the means from four independent experiments with standard errors shown as error bars.

To see the effects of the HPV oncogenes on the level of the IVL protein, we performed Western blot analysis using monoclonal antibody to IVL (Figure 3). Induction of differentiation by serum and high calcium resulted in increased IVL protein levels, as compared to that seen in proliferating cells. In proliferating cells, HPV 16 E6 or E7 alone had little effect on endogenous IVL protein, while the two oncogenes together caused strong down-regulation of IVL protein level as compared to that found in vector transduced (LXSN) cells (*p *< 0.001). This down-regulating effect of E6/E7 on IVL protein level was smaller but still significant in differentiating cells (*p *= 0.032).

### Effects of HPV 16 oncogenes on the transcriptional activity of the human *IVL *promoter

**Figure 4:**
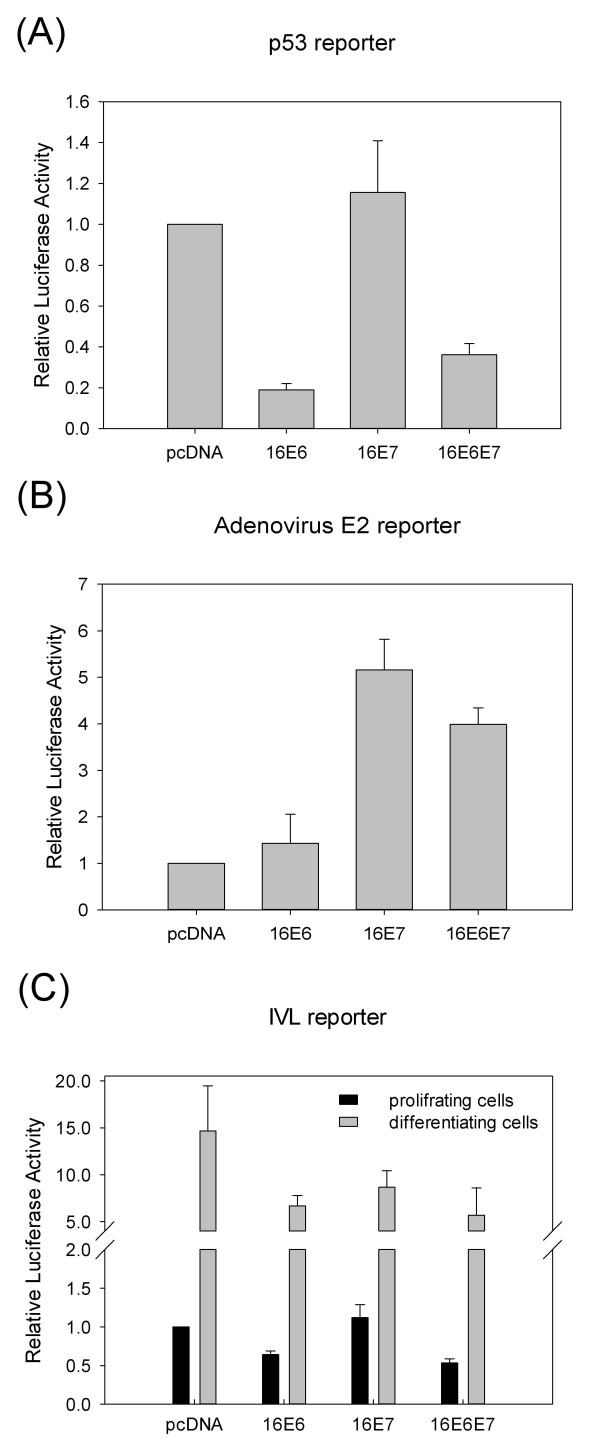
**The effect of HPV oncogenes on the transcriptional activity of different promoters in HFK cells**. (**A**) HFK cells were co-transfected with 0.5 μg of the p53 reporter construct p53-Luc, along with 0.25 μg of either control vector (pcDNA) or 0.25 μg of expression constructs encoding HPV16 E6 or E7 or both expression vectors (0.125-0.125 μg) as indicated. (**B**) HFK cells were co-transfected with 0.5 μg of the adenovirus E2 reporter construct pAdE2Luc, along with 0.25 μg of either control vector (pcDNA) or 0.25 μg of expression constructs encoding HPV16 E6 or E7 or both as indicated. (**C**) HFK cells were co-transfected with 0.5 μg of the *IVL *reporter construct pGL3-IVL, along with 0.25 μg of either control vector (pcDNA) or 0.25 μg of expression constructs encoding HPV16 E6 or E7 or both as indicated. After transfection, HFK cells were either left in serum-free medium or induced to differentiate in DMEM (with serum and high calcium). The luciferase activities are shown relative to the activity of cells co-transfected with reporter constructs and the empty expression vector (pcDNA). Data are from at least three independent experiments with standard errors shown as error bars.

Next, we aimed to determine whether the down-regulation of *IVL *expression by the HPV oncoproteins was caused by inhibiting the transcriptional activity of the *IVL *promoter. To this end, low-passage HFK cells were transiently transfected by either vector control or HPV 16 E6 and/or E7 expression vectors along with luciferase reporter constructs containing the regulatory region of the human *IVL *gene. In pilot experiments, we used an internal control vector (a *Renilla *luciferase reporter vector) to standardize for transfection efficiency. However, we found that HPV 16 E6 expression had an effect on the activity of the internal control vector. Therefore, here we show luciferase results obtained by standardization for protein concentration. The expression of functionally active HPV 16 E6 and E7 proteins in the transfected HFK cells was confirmed by co-transfection of reporter constructs containing either binding sites for the p53 protein or the adenovirus E2 (AdE2) promoter carrying binding site for the E2F transcription factor. As expected, the expression of HPV 16 E6 caused the down-regulation of the p53 reporter construct in proliferating HFK cells (Figure 4A), while HPV 16 E7 increased the activity of the adenovirus E2 promoter (Figure 4B).

In proliferating HFK cells, HPV 16 E6 caused significant down-regulation of a reporter construct (pGL3-IVL) containing the full-length regulatory region of the human *IVL *gene (*p *< 0.001). On the contrary, E7 alone had no significant effect (*p *= 0.485) on the *IVL *reporter construct in proliferating cells (Figure 4C). Induction of differentiation by serum and high calcium resulted in highly (15-fold) increased activity of the pGL3-IVL construct in the presence of the empty expression vector pcDNA. In differentiating HFK cells, both HPV 16 E6 (*p *= 0.124) and E7 (*p *= 0.253) had a non-significant tendency to inhibit *IVL *promoter activity, while HPV 16 E6 and E7 together caused significant down-regulation of the *IVL *promoter (*p *= 0.014) (Figure 4C). Taken together, these results indicate that the down-regulation of *IVL *promoter activity by HPV 16 E6 significantly contribute to the inhibition of endogenous *IVL *expression by the HPV 16 oncoproteins.

### Localisation of the effects of HPV 16 oncogenes on the human *IVL *promoter

**Figure 5:**
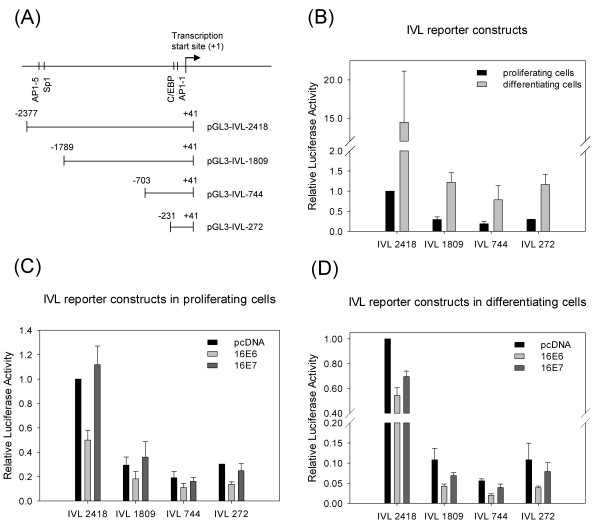
**Localisation of the effects of HPV 16 oncogenes on the human *IVL *promoter**. (**A**) Schematic representation of reporter constructs containing different parts of the human *IVL *promoter. The *IVL *promoter fragments were cloned in the reporter vector pGL3 in front of the luciferase gene. Nucleotide positions are given relative to the transcription start site. Selected transcription factor recognition sites are indicated (C/EBP, CCAAT enhancer binding protein). (**B**) The effect of differentiation on the transcriptional activity of constructs containing different fragments of the *IVL *promoter in human foreskin keratinocytes. HFK cells were co-transfected with pGL3 luciferase reporter constructs containing different fragments of the human *IVL *promoter (IVL-2418, IVL-1809, IVL-744, IVL-272) along with pcDNA control vector. Cells were left in serum-free medium or induced to differentiate in DMEM with serum and high calcium. (**C**) and (**D**) The effect of HPV 16 E6 or E7 oncogenes on the transcriptional activity of constructs containing different fragments of *IVL *promoter in proliferating (**C**) or in differentiating (**D**) HFK cells. HFK cells were co-transfected with pGL3 luciferase reporter constructs along with either control vector (pcDNA) or expression constructs encoding HPV16 E6 or E7. The luciferase activities are shown relative to the activity of cells co-transfected with IVL-2418 reporter construct along with the control vector. Data are from three independent experiments with standard errors shown as error bars.

In order to localise the effects of HPV 16 oncogenes on the human *IVL *promoter, a series of luciferase reporter vectors was constructed carrying different fragments of the promoter all extending to nt +41 relative to the transcriptional start site (Figure 5A). First, we checked in transient transfection experiments whether these constructs responded to differentiation stimuli in HFK cells. As shown on Figure 5B, the activity of each reporter construct was induced by differentiation of HFK cells. Induction by differentiation was highest (15-fold) for the longest construct (IVL-2418), while it was lower (about 4-fold) for the shorter *IVL *promoter constructs. Next, we studied the effects of HPV 16 E6 and E7 on the transcriptional activity of the IVL reporter constructs in HFK cells. In proliferating HFK cells, HPV 16 E6 was able to down-regulate transcription from each reporter construct. When compared to vector control transfected cells, E6 reduced the activity of the full-length reporter construct IVL-2418 (*p *= 0.026) and also that of the shortest reporter construct IVL-272 (*p *= 0.011) containing only the PRR (proximal regulatory region) of the *IVL *promoter (Figure 5C). E6 caused significant down-regulation of all the tested IVL reporter constructs also in differentiating HFK cells (Figure 5D). These results indicate that most of the down-regulating activity of HPV 16 E6 on the human *IVL *promoter can be mapped to the proximal regulatory region of the *IVL *gene (to the promoter region -231 to +41 relative to the transcriptional start site).

HPV 16 E7 had no significant effect on any IVL reporter construct in proliferating cells (Figure 5C). In differentiating cells, E7 had a moderate but significant inhibiting effect (*p *= 0.020) on the full-length IVL reporter construct (IVL-2418), while it had no significant effect on the shorter IVL reporter constructs.

## Discussion

In this study, we found that the HPV 16 E6 and E7 oncoproteins caused a synergistic down-regulation of endogenous IVL mRNA and protein levels in HFK cells, which are natural host cells of the virus. Our finding is in accordance with previous studies performing microarray analysis of genes involved in cervical carcinogenesis. *IVL *and/or other keratinocyte differentiation associated genes (such as certain keratins and small prolin-rich proteins) are down-regulated in cervical cancer specimens compared to normal cervical samples [20,21]. Accordingly, several studies using cultured human keratinocytes as *in vitro *models for cervical carcinogenesis found that the expression of HPV oncogenes causes a down-regulation of expression of *IVL *and/or other genes involved in epithelial differentiation [13,22,23]. However, microarray analysis does not provide information on the mechanism of changes in gene expression. Therefore, our approach was to analyse in HFK cells gene expression alterations of a few selected genes involved in keratinocyte differentiation using reliable real-time RT-PCR assays and to explore the molecular mechanisms behind gene expression alterations using luciferase reporter assays. We also found it important to study the effects of the HPV oncogenes on the expression of differentiation-regulated genes both in proliferating and in differentiating HFK cells as we thought that differentiating cells rather than proliferating cells reflect better the cellular environment required for the productive life cycle of HPV.

As expected, induction of differentiation of keratinocytes highly increased the endogenous mRNA levels of both *IVL *and *TG1 *in HFK cells (Figure 2). Interestingly, the HPV 16 E6 and E7 oncogenes together had a very strong down-regulating effect on *IVL *mRNA but only a moderate effect on *TG1 *mRNA. This suggests that the HPV oncogenes may have different effects on the expression of different genes involved in the differentiation of squamous epithelial cells. Western blot analysis showed that the joint effect of HPV 16 E6 and E7 on transcriptional down-regulation resulted in excessive decrease of IVL protein levels as well, both in proliferating and in differentiating cells (Figure 3). In a previous study, the expression of HPV 6 or HPV 16 E7 was shown to result in a decrease of IVL protein levels in HFK cells [24]. We can conclude that the HPV 16 E6 and E7 oncogenes together seem to down-regulate basal IVL expression and also decrease the differentiation-induced expression of the *IVL *gene in HFK cells.

The expression of genes involved in keratinocyte differentiation (including *IVL*) are generally regulated on the level of transcription [25]. Therefore, it seemed reasonable to investigate the effects of HPV 16 oncoproteins on *IVL *promoter activity. This approach included transfecting HFK cells by HPV 16 E6 and/or E7 expression vectors along with luciferase reporter constructs containing the whole upstream-regulatory region (URR) of the human *IVL *gene. In agreement with previous results [26], differentiation of HFK cells led to a significant increase in the transcriptional activity of the *IVL *promoter. In proliferating HFK cells, HPV 16 E6, but not E7 caused a significant down-regulation of *IVL *promoter activity. The HPV 16 E6 and E7 oncoproteins together caused a down-regulation of *IVL *promoter activity in differentiating HFK cells (Figure 4). Taken together, these results suggest that the down-regulation of endogenous *IVL *mRNA and protein levels in HFK cells by the HPV 16 E6 oncoprotein is caused by inhibition of *IVL *promoter activity. However, it can not be ruled out that HPV 16 E6 down-regulates the expression of *IVL *or other differentiation-associated genes also by other mechanisms. For example, HPV 16 E6 was shown to down-regulate the expression of Notch1, which was suggested to have a role in the suppression of keratinocyte differentiation by E6 [27].

In order to localise the effect of the HPV oncogenes within the *IVL *promoter, we made luciferase reporter constructs containing different parts of the URR of the human *IVL *gene. The URR of the human *IVL *gene contains a distal regulatory region (DRR, -2473/-1953 from transcription start site) and a proximal regulatory region (PRR, -241/-7 from the transcription start site) [26]. From the 5 possible AP1 (activator protein 1) binding sites in the URR, AP1-5 (in DRR) and AP1-1 (in PRR) are essential for optimal promoter activity [28]. AP1 factors (c-fos, fosB, Fra-1, Fra-2, c-jun, junB and junD) are expressed at specific epidermal layers and the expression pattern of these factors is thought to have a role in differentiation-regulated gene expression in keratinocytes [25,29,30]. Fra-1, junB and junD interact with AP1 sites within the human *IVL *promoter and mediate phorbol ester responsiveness [28]. In our experiments, the level of inhibition by HPV 16 E6 was the highest for the construct containing the whole URR of the *IVL *gene, but an IVL reporter construct carrying only the PRR was still significantly inhibited by the HPV 16 E6 protein, both in proliferating and in differentiating HFK cells (Figure 5). This suggests that the PRR of *IVL *gene contains binding sites for transcription factors that are regulated by HPV 16 E6.

HPV 16 E7 had a significant inhibitory effect only on the construct containing the full-length *IVL *promoter (IVL 2418), and this effect was seen only in differentiating cells (Figure 5). This may suggest that the effect of E7 on the *IVL *promoter is less direct and/or less specific than that of E6. We find it conceivable that the effects of E7 seen on IVL expression (synergistic down-regulating effect with E6) and on *IVL *promoter (slight down-regulation only in differentiating cells) are caused not by a direct and specific interaction with the *IVL *promoter, but rather by recently described other mechanisms. For instance, the DEK protein was found to be transcriptionally up-regulated by HPV 16 E7, and this was shown to be important in the induction of cell proliferation and inhibition of the epithelial differentiation program [31,32]. Furthermore, nucleophosmin (NPM) was reported to be up-regulated by HPV 16 E7 at the posttranscriptional level, and this up-regulation was suggested to have a role in the inhibition of differentiation in keratinocytes [33].

Both the DRR and the PRR of the human *IVL *gene contains binding sites for AP1 transcription factors (PRR). The promoters of differentiation-associated keratinocyte genes usually contain binding sites for the AP1 factors, and these are thought to be important in the regulation of gene expression by differentiation stimuli [25,29]. It is also interesting to note that the HPV 16 E7 protein was shown to bind to AP1 transcription factors, including c-jun, junB, junD and c-fos [34]. Therefore, we suppose that the AP1 motifs in the promoter of the human *IVL *gene may have a role in the regulation of gene expression by HPV oncoproteins. In order to prove this hypothesis, further research will be required using promoter mutagenesis and chromatin immunoprecipitation (ChIP) assays. It would be also interesting to study the effects of the HPV oncogenes on the expression of other genes (such as keratins, small prolin-rich proteins, S100 calcium binding proteins) involved in keratinocyte differentiation.

The decreased expression of IVL and other differentiation-regulated genes by the HPV oncoproteins may have an important role in the productive life cycle of the virus. HPV replication takes place in differentiating epithelial cells, which would exit the cell cycle in the absence of viral infection. The E7 oncogene is able to induce the progression of the cell cycle in differentiating keratinocytes, which is important for viral DNA replication [6,7]. On the other hand, the ability of the E6 oncogene to cause a delay in the induction of epithelial differentiation may also have a role in providing a cellular environment that is favourable for HPV replication. Our results indicate that one possible mechanism of the inhibition of keratinocyte differentiation by E6 may be the down-regulation of promoters of certain differentiation-regulated genes.

## Conclusions

In this study, the human papillomavirus 16 (HPV 16) E6 and E7 oncogenes were found to have strong synergistic inhibitory effect on the expression of endogenous IVL mRNA and protein, both in replicating and in differentiating human foreskin keratinocyte (HFK) cells. In non-differentiating HFK cells, HPV 16 E6 but not E7 down-regulated the activity of the human *IVL *promoter, and this effect could be localized to the proximal regulatory region (PRR) of the promoter. Our results indicate that the down-regulation of *IVL *promoter activity by HPV 16 E6 significantly contribute to the inhibition of endogenous *IVL *expression by the HPV 16 oncoproteins. In contrast, the down-regulation of endogenous IVL expression by HPV16 E7 is probably not caused by a direct and specific effect of E7 on the *IVL *promoter.

## Methods

### Cell culture and retroviral transduction

Primary human foreskin keratinocytes (HFK) were obtained from Invitrogen. HFK cells were cultured in Defined Keratinocyte-Serum Free Medium containing < 0.1 mM calcium (DK-SFM, Invitrogen). PA317-LXSN, -16E6, -16E7, and -16E6E7 cells are recombinant retrovirus producing cell lines from the ATCC (American Type Culture Collection). These cell lines were maintained in Dulbecco's modified Eagle's medium (DMEM) supplemented with 10% foetal calf serum, 2 mM L-glutamine and antibiotics (100 U/ml penicillin and 100 μg/ml streptomycin). Primary keratinocytes were infected with culture supernatants from PA317 cell lines producing the control LXSN virus or LXSN-based retroviral vectors expressing HPV16 E6, HPV16 E7 or HPV16 E6/E7 genes. These cells were selected in media containing G418 (100 μg/ml). Infected HFKs were either left untreated or induced to differentiate by culturing in DMEM (containing 1.8 mM calcium and 10% foetal calf serum) for 24 h.

### Plasmid constructs

The luciferase reporter vector pGL3-IVL, containing a 3.7 kb fragment of the human *IVL *gene regulatory region was described previously [35]. The other fragments of *IVL *promoter were created by PCR using pGL3-IVL as template and cloned into pGL3-Basic (Promega) between the *Xho*I and *Hind*III restriction sites resulting pGL3-IVL-2418, pGL3-IVL-1809, pGL3-IVL-744 and pGL3-IVL-272 (Table 1). Amplifications were performed with GeneAmp High Fidelity System (Applied Biosystems) according to the manufacturer's protocol. The resulting clones were verified by sequencing. The p53-Luc reporter plasmid containing several copies of the p53 binding site was purchased from Agilent Technologies. The pAdE2Luc reporter construct containing the adenovirus E2 promoter was kindly provided by Dr. Ann Roman [36]. The pcDNA-16E6 and pcDNA-16E7 expression vectors were described previously [37].

**Table 1:** Sequences of PCR primers used to construct luciferase reporter vectors containing different fragments of the human *IVL *promoter

Plasmid construct	Primer sequence (5' to 3')	Restriction site
pGL3-IVL-2418	GCGCCTCGAGCAGTGAAAGAACCTCTCCCA	*Xho*I
	GCGCAAGCTTCCATCCGACACTTACCAGAC	*Hind*III
pGL3-IVL-1809	GCGCCTCGAGATGATCCAGGAACATGACAA	*Xho*I
	GCGCAAGCTTCCATCCGACACTTACCAGAC	*Hind*III
pGL3-IVL-744	GCGCCTCGAGTTCTTATGAGCATGGCATTC	*Xho*I
	GCGCAAGCTTCCATCCGACACTTACCAGAC	*Hind*III
pGL3-IVL-272	GCGCCTCGAGAGTTGAGCTACCAGAATCCT	*Xho*I
	GCGCAAGCTTCCATCCGACACTTACCAGAC	*Hind*III

PCR products amplified using the indicated primers were digested by *Xho*I and *Hind*III and cloned into the luciferase reporter vector pGL3

### Transient transfection

Primary human keratinocytes (within 3-6 passages) were plated on 6-well plates at approximately 80% confluence. The cells were co-transfected by 0.5 μg of *IVL *promoter-luciferase constructs (pGL3-IVL, pGL3-IVL-2418, pGL3-IVL-1809, pGL3-IVL-744 or pGL3-IVL-272) along with 0.25 μg of expression vectors (pcDNA) encoding HPV 16 E6 and/or E7 genes using Effectene (Qiagen). The total amount of expression vectors was kept constant (0.25 μg) in all transfection experiments. Transfection mix was added to cells in Opti-MEM (Invitrogen) and incubated for 5 h at 37°C, after which the medium was changed to DK-SFM. Twenty-four hours after transfection, HFKs were either left untreated or induced to differentiate in DMEM (containing 1.8 mM calcium and 10% foetal calf serum for 24 h). The cells were washed with PBS (phosphate buffered saline) 48 h after transfection and lysed in Reporter Lysis Buffer (Promega). A Berthold luminometer and Luciferase assay system (from Promega) was used to measure luciferase activity. Bradford protein assay was performed to standardize for the protein concentration of the cell extracts. Each transfection experiment was performed independently at least three times.

### RT-PCR

Total RNA was isolated from proliferating or differentiating transduced cells by using TRI reagent (Sigma). The High Capacity cDNA Reverse Transcription Kit (Applied Biosystems) was used to prepare cDNA. The PCR reaction was performed with GoTaq DNA polymerase (Promega) according to the manufacturer's protocol. The primer pairs used for amplifying HPV16 E6, E7 and GAPDH (glyceraldehyde 3-phosphate dehydrogenase) were previously described [38].

### Real-time RT-PCR

After total RNA isolation, cDNA was synthesised as described above. The real-time PCR was performed on the 7500 Real Time PCR System (Applied Biosystems) using TaqMan Gene Expression Master Mix and Assays according to the manufacturer's recommendations (Applied Biosystems). The PCR amplification was carried out in a total volume of 20 μl. The TaqMan Gene Expression Assays used were for involucrin (IVL; Hs00846307_s1), transglutaminase 1 (TG1; Hs00165929_m1), cyclin-dependent kinase inhibitor 2A (CDKN2A, p16-INK4A; Hs00923894_m1), telomerase reverse transcriptase (TERT; Hs00162669_m1) and glyceraldehyde 3-phosphate dehydrogenase (GAPDH; 0711024) as endogenous control (Applied Biosystems). Each PCR reaction was performed in triplicate at least three times.

### Western blot

Protein extract from proliferating or differentiating transduced HFK cells were prepared in RIPA lysis buffer (150 mM NaCl, 1% NP-40, 50 mM Tris-HCl pH 8.0, 0.5% Na-dezoxycholate, 0.1% SDS, 0.01% Na-azide, 1 mM EDTA, pH 7.4) supplemented with Complete EDTA-free Protease Inhibitor Cocktail (Roche). Cells were scraped, incubated on ice, and after centrifugation the supernatants were collected. The protein concentration of the lysates was measured by Bradford protein assay. Ten μg of protein extracts was electrophoresed through 10% polyacrilamide gels (SDS-PAGE) and electroblotted into nitrocellulose membrane. Membranes were blocked in Tris buffered saline-Tween (TBST: 10 mM Tris, 150 mM NaCl, 0.05% Tween 20, pH 7.9) containing 5% non-fat dry milk. The blots were incubated with primary antibodies overnight at 4°C. The following primary antibodies were diluted 1:2000 in 5% non-fat dry milk in TBST: mouse monoclonal anti-involucrin (sc-56555), mouse monoclonal anti-p53 (sc-126) from Santa Cruz Biotechnology and rabbit polyclonal anti-actin (A2066) from Sigma. After washing in TBST, the membrane was incubated for 1 h with goat anti-mouse (sc-2005) or goat anti-rabbit (sc-2004) secondary antibodies conjugated with horseradish peroxidase (Santa Cruz Biotechnology) diluted 1:5000 in 5% non-fat dry milk in TBST. Following washes in TBST, antibody complexes were visualized using the SuperSignal West Pico Chemiluminescent Substrate (Pierce) and exposed to X-ray films. The amounts of proteins were quantitatively determined by densitometry using Gel Doc 2000 gel documentation system (Bio-Rad) and the Quantity One (version 4.0.3) software. Protein levels of IVL and p53 were normalized to actin levels.

### Statistical analysis

For the analysis of real-time RT-PCR results, the comparative Ct method was used to obtain the Relative Quantification (RQ) values with standard deviation and confidence intervals (7500 System SDS Software, version 1.4). To analyse the results of luciferase tests, mean and SEM (standard error of mean) of standardized luciferase values (from at least 3 independent experiments) were calculated, and the significance of differences between 2 mean values was evaluated using the 2-sample *t*-test. Significance was accepted at *p *< 0.05.

## Abbreviations

AP1: activator protein 1; CCE: cornified cell envelope; CDKN2A: cyclin-dependent kinase inhibitor 2A; C/EBP: CCAAT enhancer binding protein; ChIP: chromatin immunoprecipitation; DK-SFM: Defined Keratinocyte-Serum Free Medium; DMEM: Dulbecco's modified Eagle's medium; DRR: distal regulatory region of the human involucrin gene; EDTA: ethylenediaminetetraacetic acid; GAPDH: glyceraldehyde 3-phosphate dehydrogenase; HFK: human foreskin keratinocyte; HPV: human papillomavirus; IVL: involucrin: NPM: nucleophosmin; p16INK4A: cyclin-dependent kinase inhibitor 2A protein; PBS: phosphate buffered saline; pRB: retinoblastoma protein; PRR: proximal regulatory region of the human involucrin gene; RQ: relative quantification; SDS: sodium dodecyl sulfate; SEM: standard error of mean; SPRR: small praline rich proteins; TBST: Tris buffered saline-Tween; TERT: telomerase reverse transcriptase; TG1: transglutaminase 1; URR: upstream regulatory region of the human involucrin gene.

## Competing interests

The authors declare that they have no competing interests.

## Authors' contributions

EG carried out the majority of experiments and participated in drafting the manuscript. AS participated in the optimization of the methods. AF took part in cloning the reporter constructs and optimisation of transfection. JK and LG participated in the design of the study and in data analysis. VG conceived and designed the study, produced the retrovirus vector transduced cell lines and helped to draft the manuscript. All authors have read and approved the final version of the manuscript.
